# Supplementary figures and images for: Describe the morphology and mitochondrial genome of Mecidea indica Dallas, 1851 (Hemiptera, Pentatomidae), with its phylogenetic position
Source: PLoS One. 2024 Mar 28;19(3):e0299298. doi: 10.1371/journal.pone.0299298 (PMC10977800; doi:10.1371/journal.pone.0299298)

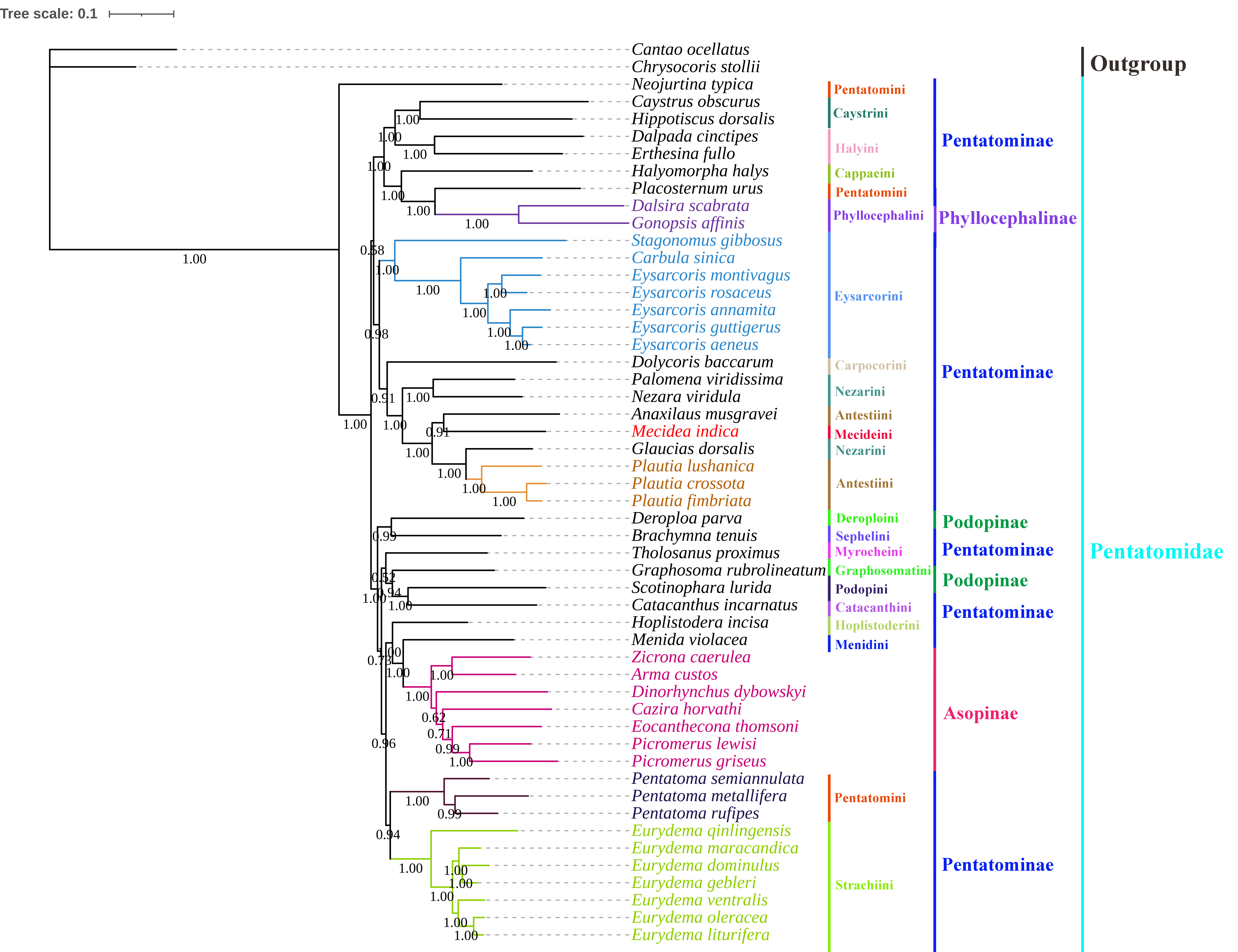

Supplement: S1 Fig — The number on the branches indicates Bayesian posterior probabilities. (TIF) [file pone.0299298.s001.tif]
