# Supplementary material for: Describe the morphology and mitochondrial genome of Mecidea indica Dallas, 1851 (Hemiptera, Pentatomidae), with its phylogenetic position
Source: PLoS One. 2024 Mar 28;19(3):e0299298. doi: 10.1371/journal.pone.0299298 (PMC10977800; doi:10.1371/journal.pone.0299298)
Supplement: Attachment — Submitted filename: Response to Reviewers.doc [file pone.0299298.s004.doc]

Dear Editor,

Thanks for your letter and for reviewers’ comments concerning our manuscript entitled “Describe the morphology and mitochondrion genome of *Mecidea indica* Dallas, 1851 (Hemiptera, Pentatomidae), with its phylogenetic position”. These comments are all valuable and helpful for revising and improving our paper. We have studied all comments carefully, and have made conscientious correction. Revised portion are marked in red in the paper. The main corrections in the paper and the responds to the reviewers’ comments are as flowing.

Thank you for your consideration. I look forward to hearing from you.

Sincerely

Qing Zhao

**Response to Reviewer #1**

Point 1: The discussion may be strength.

**Response:** Thank you very much for your comments on my article, we have made corresponding modifications. We have revised the Discussion accordingly.

Point 2: The language may be improved by a native English speaker.

**Response:** We have improved our manuscript by Editage service, and the polishing proof of this manuscript is provided.

Point 3: Evidence of animal ethics needs to be added in manuscript.

**Response:** The species we used for scientific purposes is not protected animals and meet animal ethical requirements. It is ethical, humane and responsible. And we have added the sentence in our manuscript.

Point 4: The figures are not clear.

**Response:** Thank you very much for your comments on my article. We have processed the figures.

Point 5: Line 80 some new paper you can cite such as: https://doi.org/10.1038/s41598-022-17814-8;

DOI: 10.3724/ahr.2095-0357.2023.0003.

**Response:** Thank you very much for your comments on my article, we have made corresponding modifications.

**Response to Referee #2**

Point 1: In this paper, the authors checked a series of specimens, so the species measurements should be a range rather than a fixed value.

**Response:** You are right that the measurement should be a numerical interval, which we have already added.

Point 2: In “Materials and Methods”, What was the concentration of DNA? Did you use total DNA for sequencing? How the libraries were prepared and how the quality of the libraries was estimated? How did you perform de novo genome assembly in Genious and how did you determined the mitochondrial contigs?

**Response:** Thank you very much. We used fluorescent dye (Quant it PicoGreen dsDNA Assay Kit) to detect the total amount of DNA. The total amount of DNA was 2.39 μg, and the fluorescence concentration was 47.80 ng/μl. After passing the quality inspection, the required genomic library was constructed using the standard Illumina TruSeq Nano DNA LT library preparation experimental process (Illumina TruSeq DNA Sample Preparation Guide). We chose “Map to Reference” for assembly instead of “De Novo Assemble”. And we used “HQ%” to determine the mitochondrial contigs.

Point 3: It needs a scale bar on the Figure 1 about the two adult figures.

**Response:** Thank you for your advice, we have added the scale bar.

Point 4: Instead of providing many trees, please give one which summarize all your conclusions. All other trees as well as many other graphical data from your research could be given in Supplement.

**Response:** Thank you very much for your comments on my article. We had chosen a phylogenetic tree based on the PRT dataset with higher bootstrap values to represent our results. And the other phylogenetic tree was placed in Supplement.

Point 5: The description of genes should be consistent throughout the text, such as cob in some places and cytb in others.

**Response:** Thank you very much for your comments on my article, we have made corresponding modifications.

Point 6: Which model was determined by PartitionFinder? Is this model implemented in MrBayes?

**Response:** Thank you very much for your comments on my article. The model of Partition Finder was placed in Supplement. The model is implemented in MrBayes.

Point 7: The writing and language is not up to standard. Please proofread and use the spell checking functions in the word processing software of choice before submitting manuscripts.

**Response:** Thank you, we have accepted the advice and corrected it.

**Response to Referee #3**

Reviewer #3: In this study, the author described external morphology and the complete mitochondrial genome characteristics of *Mecidea indica*. Moreover, the author clarified the evolutionary rate and divergence time of *M. indica* and discussed the phylogenetic relationships of tribes within the family Pentatomidae. At present, the classification of tribes and subfamilies of Pentatomidae is still based on traditional taxonomic studies, this study is important for reconstructing the phylogeny of Pentatomidae and understanding the evolutionary history of Pentatomidae.

**Response:** Thank you very much for your comments on our manuscript.
