# Supplementary material for: Describe the morphology and mitochondrial genome of Mecidea indica Dallas, 1851 (Hemiptera, Pentatomidae), with its phylogenetic position
Source: PLoS One. 2024 Mar 28;19(3):e0299298. doi: 10.1371/journal.pone.0299298 (PMC10977800; doi:10.1371/journal.pone.0299298)
Supplement: Attachment — Submitted filename: Response to Reviewers.doc [file pone.0299298.s005.doc]

Dear Editor,

Thanks for your letter and for reviewers’ comments concerning our manuscript entitled “Describe the morphology and mitochondrion genome of *Mecidea indica* Dallas, 1851 (Hemiptera, Pentatomidae), with its phylogenetic position”. These comments are all valuable and helpful for revising and improving our paper. We have studied all comments carefully, and have made conscientious correction. Revised portion are marked in red in the paper. The main corrections in the paper and the responds to the reviewers’ comments are as flowing.

Thank you for your consideration. I look forward to hearing from you.

Sincerely

Qing Zhao

**Response to Reviewer #3**

Point 1: The format of the numbers should be consistent, for example: “15,670 bp” on line 33 is different from “15670 bp”on line 323.

**Response:** Thank you very much for your comments on my article, we have made corresponding modifications.

Point 2: Point 2: In line 202, “95%HPD” should be “95% HPD”.

**Response:** We have improved our manuscript by Editage service, and the polishing proof of this manuscript is provided.

Point 3: The abbreviation "protein-coding genes (PCGs)" should appear when "protein-coding genes" first appears, and it should not be repeated later, for example: in line 174 and line 324.

**Response:** Thank you very much for your comments on my article, we have made corresponding modifications.
